# A Call to Action: Current Challenges and Considerations for COVID-19 Vaccination in Immunocompromised Populations

**DOI:** 10.1093/infdis/jiad150

**Published:** 2023-08-04

**Authors:** Paolo Bonanni, Francesca Ceddia, Rachel Dawson

**Affiliations:** Department of Health Sciences, University of Florence, Florence, Italy; Moderna, Inc., Cambridge, Massachusetts, USA; Moderna, Inc., Cambridge, Massachusetts, USA

**Keywords:** COVID-19, immunocompromised, vaccination

## Abstract

The heightened risk of infection and complexities of preventing disease in immunocompromised individuals are at the forefront of public health strategies. The COVID-19 pandemic highlighted the increased vulnerability and susceptibility to serious outcomes in this population. COVID-19 prevention efforts led to the development of vaccines, including mRNA-based options, which were initially recommended as a 2-dose primary schedule for both immunocompromised and immunocompetent individuals. However, post-rollout assessments led to updated recommendations specific to immunocompromised populations. As COVID-19 potentially transitions to become endemic disease, immunocompromised individuals will remain at high risk of severe disease; thus, the evaluation of current vaccination challenges remains crucial for guiding effective public health efforts. This article summarizes key findings from the previous articles of this supplement, highlighting current vaccination challenges for at-risk immunocompromised groups and exploring solutions to ensure protection against COVID-19 for these vulnerable populations.

## THE CONSTANT YET EVOLVING RISK FOR COVID-19 AMONG IMMUNOCOMPROMISED POPULATIONS

The high risk of infection among individuals with compromised immune systems as well as the complexities of preventing and mitigating infectious disease in these patients are at the forefront of public health strategies. The unique challenges facing immunocompromised patients were exemplified during the global COVID-19 pandemic, wherein it quickly became apparent that these individuals were particularly vulnerable to serious outcomes [1].

The urgent need for preventive measures against SARS-CoV-2, the causative pathogen of COVID-19, led to the unprecedented development of multiple COVID-19 vaccines, including those based on novel mRNA technology (mRNA-1273 [SPIKEVAX; Moderna, Inc., Cambridge, MA, USA] and BNT162b2 [COMIRNATY; Pfizer Inc., New York, NY, USA; BioNTech Manufacturing GmbH, Mainz, Germany]). These mRNA-based vaccines were initially recommended for use in immunocompromised populations as a 2-dose primary regimen, similar to immunocompetent populations. However, characterizations of vaccine-elicited immunogenicity after vaccine rollout indicated that a 2-dose primary schedule was likely insufficient for these populations, resulting in updated recommendations specific to immunocompromised populations: first to an extended 3-dose primary schedule, and then for additional doses to further boost immune responses [2, 3].

As SARS-CoV-2 continues to evolve into new, increasingly transmissible variants of concern and the pandemic potentially transitions into a more endemic state, susceptibility to severe disease will remain high for high-risk populations, such as immunocompromised individuals. Thus, acknowledging, understanding, and tackling the current challenges of vaccination across the diverse populations of immunocompromised patients remains an important public health need and is critical for guiding effective healthcare efforts for this population. In this concluding article of the supplement, we summarize the key findings of the previous articles describing COVID-19 vaccination in heterogeneous immunocompromised populations, describe the current challenges facing vaccination strategies in these risk groups, and consider how to address hurdles and ensure that these vulnerable populations are protected against COVID-19.

## COVID-19 VACCINATION ACROSS THE SPECTRUM OF IMMUNOCOMPROMISED POPULATIONS

The previous articles in this supplement provided an in-depth characterization of mRNA-based COVID-19 vaccination across the heterogeneous populations of immunocompromised individuals, including the following: patients with rheumatic disease and immune-mediated inflammatory disease, primary immunodeficiency, and solid organ transplants; patients receiving dialysis; and patients with cancer or who are receiving hematopoietic stem cell transplant or chimeric-antigen receptor T-cell therapy.

It is notable that responses to vaccination vary depending on the immunocompromised patient population and the cause of immunosuppression. If the cause of immunosuppression is due to treatment for an underlying disease, the type of immunosuppressive therapy received will dictate vaccination response, with B cell–depleting therapies such as rituximab known to greatly reduce vaccine-elicited immune responses. More importantly, regardless of the cause of immunosuppression, additional doses of mRNA-based COVID-19 vaccines can increase antibody responses. Nevertheless, some immunocompromised patients remain nonresponsive to additional vaccine doses, which warrants further investigation. Taken together, COVID-19 vaccination approaches have appreciable effects among the immunocompromised, but the lack of homogeneity within the immunocompromised populations has important implications for ongoing vaccination efforts in these patients.

## ADDRESSING THE CURRENT CHALLENGES OF COVID-19 VACCINATION AMONG IMMUNOCOMPROMISED POPULATIONS

**Figure 1.**
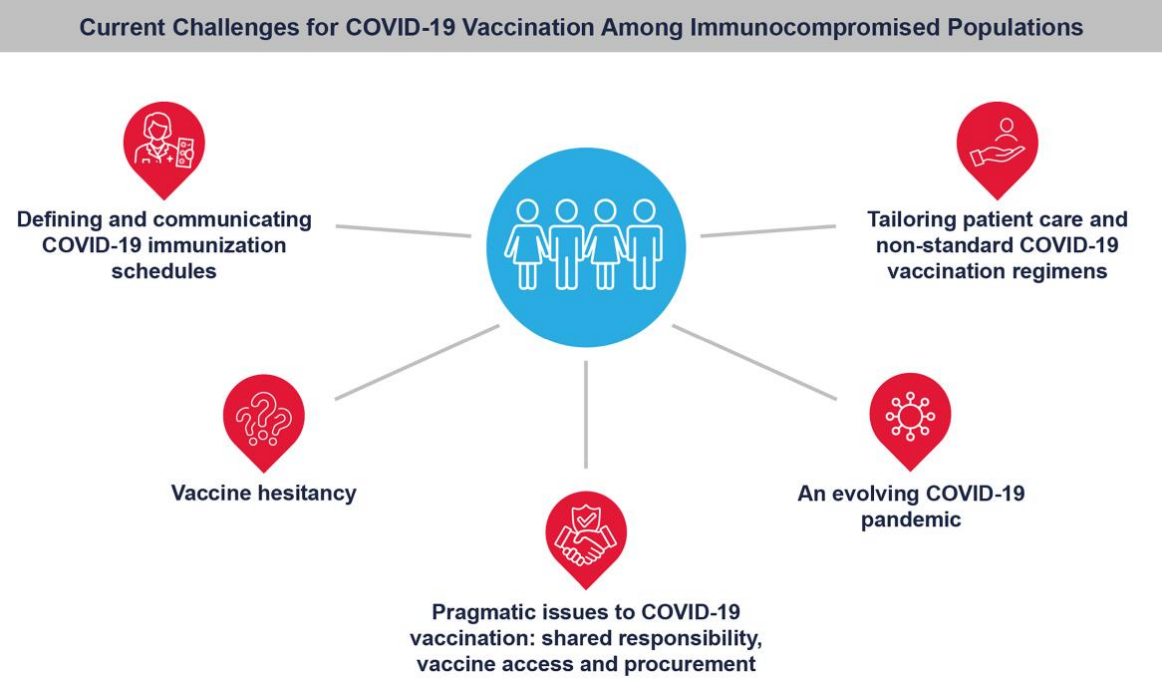
Current challenges for COVID-19 vaccination among immunocompromised populations.

Safely and effectively immunizing immunocompromised populations against infectious disease is inherently challenging, but overcoming these hurdles while navigating the convoluted public health landscape of the COVID-19 pandemic raised further challenges for this population. In this supplement, we describe the challenges facing successful immunization of immunocompromised patients and discuss key approaches toward overcoming these barriers to further mitigate disease burden among these populations (Figure 1).

### Defining and Communicating Immunization Schedules

Throughout the COVID-19 pandemic, vaccination schedules have been amended in real time for immunocompromised populations to enable up-to-date guidance in a dynamic public health environment. In the United States, mRNA-based COVID-19 vaccines were first authorized as a 2-dose primary regimen for emergency use among adults (aged ≥16 or 18 years) in December 2020, similar to immunocompetent individuals [4, 5]. However, immunization guidelines were amended in August 2021 to allow for an additional primary (third) dose in the mRNA COVID-19 vaccination series among immunocompromised individuals, followed by further amendments in September–October 2021 to allow for a single booster (fourth) dose of an mRNA-based vaccine ≥6 months after completing the primary series [6]. Guidelines were again amended in March 2022 to allow for a second booster (fifth) dose of an mRNA-based vaccine ≥4 months after the first booster for immunocompromised individuals [7]. Most recently, in September 2022, bivalent formulations of mRNA-based vaccines were authorized as a single booster dose ≥2 months after primary or booster vaccination [8]. As of this writing, current US guidelines for mRNA-based vaccines for moderately or severely immunocompromised adults are for a 3-dose primary series of the monovalent vaccine (with ≥4-week intervals between doses 2 and 3) and a single booster dose of a bivalent vaccine administered ≥2 months after the primary series [9].

Immunization schedules for immunocompromised patients during the COVID-19 pandemic have evolved to ensure populations remain continuously protected; however, the multiple modifications may have collateral impacts on adherence and vaccine uptake among this population. For example, a recent retrospective cohort study of Kaiser Permanente Southern California patients from December 2020 to August 2022 indicated that although 78.0% of immunocompromised participants received a third dose of an mRNA COVID-19 vaccine, only 41.0% and 0.9% had received a fourth and fifth dose, respectively [10]. The reason for the low uptake of the fourth and fifth doses in immunocompromised individuals is unclear. However, the low uptake of additional vaccine doses may be due to confusion surrounding the definitions of the primary series, booster doses, and what constitutes being “up-to-date” with vaccine recommendations for immunocompromised populations rather than vaccine hesitancy [10].

Other factors beyond confusion surrounding vaccine recommendations may also impact vaccine uptake among immunocompromised individuals. It is possible that the increasing availability of antivirals and monoclonal antibodies as therapeutic options for the treatment of COVID-19 reduced the urgency to get vaccinated [11]. In addition, the lack of understanding of long-term side effects, anxiety toward possible allergic reactions, and skepticism regarding the effectiveness of the vaccine are all factors that can affect uptake among immunocompromised populations [12]. Another consideration affecting COVID-19 vaccine uptake is the use of certain immunomodulatory medications that have the potential to impact vaccine-mediated immune responses thereby limiting the long-term protection against COVID-19. As a result, healthcare providers may advise individuals with rheumatic disease or immune-mediated inflammatory disease to temporarily stop specific immunosuppressive medications before receiving the vaccine [13]; however, caution should be taken because this may increase the risk of flare ups in the underlying inflammatory condition.

As policymakers begin to consider integrating COVID-19 vaccination into immunization programs and potentially implementing an annual COVID-19 booster vaccination, they are encouraged to use definitive language such as “yearly booster.” Such alignment on the immunization schedule may simplify vaccination for healthcare professionals and their immunocompromised patients, removing confusion and allowing for targeted and sustained protection in vulnerable populations. In addition, by improving healthcare professional awareness of COVID-19 immunization schedules and ensuring that their patients are aware of the recommended schedule for vaccination, we may help to improve vaccine uptake.

### Tailoring Patient Care and Nonstandard Vaccination Regimens

The COVID-19 vaccination guidelines for immunocompromised individuals provided by the Centers for Disease Control and Prevention [14] (Table 1), the European Medicines Agency, and the National Health Service are fairly aligned, all recommending a third primary dose for this population [15]. Alignment on nonstandard vaccination regimens, such as administering a higher dose, is also a priority [14, 16]. Moving forward, it is imperative that the experts in charge of developing COVID-19 immunization recommendations are cognizant of the heterogeneity of immunocompromised patient populations and their disparate immune responses to vaccination. In this regard, further characterization of the magnitude, breadth, and durability of vaccine-elicited immune responses in each patient group is warranted to further identify those patients who require nonstandard vaccination approaches. Continued clinical, observational, and real-world investigation of the safety and immunogenicity of mRNA-based COVID-19 vaccines in distinct patient populations can aid in further informing immunization schedules to ensure sufficient protection. In addition, ongoing guidance and outreach to patients, healthcare professionals, and other key stakeholders on emerging scientific and medical knowledge of COVID-19 vaccination for patient groups will remain essential to increase awareness of vaccination options and opportunities.

**Table 1.** mRNA-Based COVID-19 Vaccine Recommendations for Immunocompromised Individuals in the United States

	Age Group			
Vaccine Series/Dose	6 Months–4 Years	5 Years	6–11 Years	≥12 Years
mRNA-1273				
Primary series dose 1	25 µg	25 µg	50 µg	100 µg
Interval between dose 1 and 2	28 d	28 d	28 d	28–56 d
Primary series dose 2	25 µg	25 µg	50 µg	100 µg
Interval between dose 2 and 3	28 d	28 d	28 d	28–56 d
Primary series dose 3	25 µg	25 µg	50 µg	100 µg
Interval between dose 3 and booster	60 d	60 d	60 d	60 d
Booster dose	10 µg^a^	10 µg^a^	25 µg^a^	50 µg^a^
BNT162b2				
Primary series dose 1	2 µg	10 µg	10 µg	30 µg
Interval between dose 1 and 2	21–56 d	21–56 d	21–56 d	21–56 d
Primary series dose 2	2 µg	10 µg	10 µg	30 µg
Interval between dose 2 and 3	56 d	28–56 d	21–56 d	21–56 d
Primary series dose 3	2 µg^a^	10 µg	10 µg	30 µg
Interval between dose 3 and booster	60 d	60 d	60 d	60 d
Booster dose	2 µg^a^	10 µg^a^	10 µg^a^	30 µg^a^

Abbreviations: d, days.

Bivalent.

Given the heterogeneity of the immunocompromised population, adoption of nonstandard vaccination approaches could help to provide tailored patient care for those with attenuated responses to vaccination. When providing guidelines for immunization recommendations, the Centers for Disease Control and Prevention considers timing of the COVID-19 vaccination as it relates to immunosuppressive therapies [17]. For COVID-19, the current guidance for this population is to wait ≥2 weeks before starting or resuming immunosuppressive therapy after COVID-19 vaccination. Those patients receiving B cell–depleting therapies are advised to time their vaccination to coincide with approximately 4 weeks before their next scheduled therapy. Currently, US healthcare professionals have the potential to administer COVID-19 vaccines outside of the recommended dosing intervals if there are any clinical benefits of doing so; however, nonroutine vaccinations are not recommended as a standard practice [17]. Active postvaccination assessment of immune response to vaccination through serologic, cellular, immune, or B cell quantification testing is currently not recommended to guide clinical care, although this approach may ultimately be important to establish for COVID-19 vaccines. For example, serologic tests have been used for assessing the need for hepatitis B revaccination or booster doses among hemodialysis patients [18]; whether a similar approach might be practical for COVID-19 vaccines among immunocompromised patients may be worth further investigation. Overall, alignment on recommendations for tailored care may help to simplify immunization options, increase patient care options, and ultimately provide additional protection for those individuals currently still at risk for serious outcomes from SARS-CoV-2 infection.

### Vaccine Hesitancy

Although studies have shown that the majority of immunocompromised patient populations are accepting of COVID-19 vaccination, some hesitancy among these vulnerable individuals has also been identified. A cross-sectional, internet-based survey of approximately 22 000 individuals found that hesitancy was expressed by 13.4%, 19.4%, and 17.8% of patients with cancer, autoimmune diseases, and chronic lung diseases, respectively [19]. Among patients with primary immunodeficiency in Canada, 18.4% (68 of 376 survey respondents) were undecided, somewhat/very unlikely, or not planning to receive vaccination [12]. Although the underlying factors for vaccine hesitancy among the immunocompromised are multifaceted, patient uncertainty of the benefit of vaccination given their immunocompromised status has been noted as a primary cause [12, 19]. In addition, perceptions of safety concerns as well as the lack of information on vaccine effectiveness and side effects remain notable reasons, which are compounded by the fact that immunocompromised populations were excluded from the initial vaccine clinical trials [12, 19]. General distrust of COVID-19 vaccine development has also been identified, a factor known for vaccine hesitancy among the general population as well [12, 19].

Strategies to increase confidence and acceptance of COVID-19 vaccination among immunocompromised populations therefore remain important. It is essential for patients and providers to understand that mRNA-based COVID-19 vaccines have shown no major safety concerns among immunocompromised populations [14]. Based on safety reports received by v-safe and the Vaccine Adverse Event Reporting System (VAERs) between January and March 2022, serious adverse events among immunocompromised individuals aged ≥12 years were rare after the first booster (fourth) dose of an mRNA COVID-19 vaccine [20]. In general, the biggest potential risk among this population is decreased vaccine effectiveness against disease; thus, the benefit versus risk of vaccination among immunocompromised patients is potentially even more obvious than for immunocompetent individuals who are less susceptible to severe COVID-19 outcomes.

It is notable that healthcare professionals can have a particularly impactful role on reducing vaccine hesitancy, with recommendations for vaccination from a professional allaying concern to thereby increase likelihood of vaccine receipt. In a study examining barriers to COVID-19 vaccination among adults with immune-mediated inflammatory disease, concerns over vaccination-associated, immune-mediated, inflammatory disease flares (a key barrier to vaccination) were found to dissipate after patient discussion with a trusted healthcare professional [21]. Thus, ensuring that healthcare professionals (specialists and primary care physicians) of immunocompromised patients are aware of current immunization recommendations and are knowledgeable of the potential benefits and limitations of these vaccines in each population may help to alleviate concerns and reduce overall hesitancy.

### An Evolving COVID-19 Pandemic

Throughout the COVID-19 pandemic, evolving SARS-CoV-2 variants with increased transmissibility and escape from infection- or vaccine-induced immunity has been a constant risk. Most notably, the omicron variant lineage (B.1.1.529 [including BA.1, BA.2, BA.4, and BA.5]) emerged in November 2021, with substantial mutations to the antigenic S protein [22]. Among immunocompetent populations, vaccine-elicited neutralizing activity and overall vaccine effectiveness against omicron infection remained lower relative to those against ancestral SARS-CoV-2 or other earlier variants [23, 24]. The omicron variant poses similar concerns for adults with immunocompromising conditions such as cancer, inflammatory diseases, immunodeficiency, organ or stem cell transplant, and chronic respiratory diseases, with vaccine effectiveness estimates against COVID-19-associated hospitalization during the predominant period of omicron (BA.1) of 36% (2-dose regimen), 67% (≥7 days after dose 3), 32% (≥90 days after dose 3), and 43% (7 days after dose 4) [25]. Accordingly, vaccination strategies have adapted to broaden protection against SARS-CoV-2 variants, including omicron or other potential variants of concern, that emerge as the virus continues to evolve.

Currently, bivalent mRNA-based vaccines containing equal amounts of ancestral and omicron (BA.1 or BA.4/BA.5) strains are now authorized as booster doses in multiple countries worldwide, including for use among immunocompromised populations [8]. Because limited data are currently available for the use of bivalent vaccines in immunocompromised populations, careful monitoring of the immunogenicity and effectiveness of these vaccines in these disparate populations will be required. In addition, as mentioned earlier, ensuring that healthcare professionals and patients are aware of the evolving immunization recommendations for COVID-19 vaccines will be essential toward ensuring vaccine uptake, especially because SARS-CoV-2 continues to evolve and an annual booster vaccination is being considered in countries such as the United States.

In addition to the bivalent mRNA-based booster doses, updated COVID-19 monoclonal antibodies could potentially be used as a prophylactic treatment for immunocompromised individuals who may not mount an adequate immune response to COVID-19 vaccination [26]. Preliminary studies suggest that prophylaxis with monoclonal antibodies may help reduce infection rates and limit viral circulation, potentially preventing the selection of variants [26, 27]. However, further studies are needed to verify the effectiveness of this strategy.

### Pragmatic Issues to Vaccination

With the integration of COVID-19 vaccination into immunization programs worldwide and the implementation of annual COVID-19 booster vaccination under discussion, there remain certain pragmatic challenges. At the forefront is a need to standardize vaccination responsibilities among healthcare professionals (eg, primary care providers or specialists). Although specialists are intimately involved in specialized care for an immunocompromised patient, these professionals often perceive that immunizations instead fall within the purview of primary care providers [28]. However, certain patient populations might receive care primarily from a specialist as opposed to a primary care provider, thus missing opportunities for vaccination [29]. As such, recommendations from national policymakers or additional guidelines from professional societies are needed to define responsibilities among healthcare professionals to ensure immunocompromised populations have informed discussions on vaccination and are aware of vaccination opportunities. Of note, the Infectious Diseases Society of America published guidelines in 2013 recommending that specialists share vaccination responsibilities with primary care providers for immunocompromised patients and their household members [30]. Beyond standardizing these definitions, ensuring that healthcare professionals are knowledgeable of both the basic aspects of COVID-19 vaccination as well as the intricacies of vaccination for particular immunocompromising conditions or therapies (such as optimal timing) will be required to optimize protection for these groups. As discussed previously, the attitudes of healthcare professionals towards vaccination can greatly influence vaccination decisions among their patients, but it requires these professionals to understand and adhere to current immunization recommendations.

Vaccine access and procurement will also remain a pragmatic challenge for vaccination of at-risk immunocompromised populations, particularly if SARS-CoV-2 becomes endemic. By 2026, global economic losses could exceed an estimated 5.3 trillion US dollars if the virus becomes endemic [31]. Despite the method of procurement for these vaccines, governments will have to take into consideration the additional number of doses needed for immunocompromised populations when planning future vaccine needs. Equitable vaccine access also remains a major challenge, with the World Health Organization stating vaccine coverage ranged from 1% to >70% in 2021 due primarily to nation wealth [31]. Lack of vaccine access can spur disease surges and potentially allow for SARS-CoV-2 variants to emerge, because persistent infections among immunocompromised individuals can generate variants with increased transmissibility or pathogenicity [32]. Two upcoming changes in the United States have the potential to affect the pricing, coverage, and, ultimately, the access to COVID-19 vaccines among the immunocompromised population. First, the depletion of the federally purchased supply of COVID-19 vaccines is expected to result in a transition to the commercial market for manufacturing, procurement, and pricing. Second, the public health emergency declaration ended on May 11, 2023 [33]. Taken together, these changes may lead to increased cost sharing, as well as limited coverage and supply, which could adversely affect vaccine access for the immunocompromised population in the United States. Therefore, ensuring resource allocation for vaccinating immunocompromised populations and equitable access to vaccines remain essential as the COVID-19 pandemic continues to evolve and potentially transitions into an endemic state.

## CONCLUSIONS

Throughout the COVID-19 pandemic, vaccination has been an essential strategy to mitigate disease burden among immunocompromised individuals. The aim of the articles in this supplement has been to summarize the vaccination of at-risk immunocompromised populations for COVID-19, describe the difference in response to COVID-19 vaccines in these populations, and consider the limitations and benefits of current vaccination strategies in these populations. By understanding and addressing the challenges facing these heterogeneous populations, the medical and public health communities can sustain momentum and optimize disease prevention as the pandemic continues to progress and potentially becomes endemic.
